# Ultrafast nonlinear optical properties of thin-solid DNA film and their application as a saturable absorber in femtosecond mode-locked fiber laser

**DOI:** 10.1038/srep41480

**Published:** 2017-01-27

**Authors:** Reza Khazaeinezhad, Sahar Hosseinzadeh Kassani, Bjorn Paulson, Hwanseong Jeong, Jiyoon Gwak, Fabian Rotermund, Dong-Il Yeom, Kyunghwan Oh

**Affiliations:** 1 Photonic Device Physics Laboratory, Institute of Physics and Applied Physics, Yonsei University, Seoul 120–749, South Korea; 2 Harvard Medical School, Boston, Massachusetts 02115, USA; 3 Wellman Center for Photomedicine, Massachusetts General Hospital, Boston, Massachusetts 02114, USA; 4 Department of Physics & Energy Systems Research, Ajou University, Suwon 443-749, South Korea; 5 Department of Physics, Korea Advanced Institute of Science and Technology, Daejeon 34141, South Korea

## Abstract

A new extraordinary application of deoxyribonucleic acid (DNA) thin-solid-film was experimentally explored in the field of ultrafast nonlinear photonics. Optical transmission was investigated in both linear and nonlinear regimes for two types of DNA thin-solid-films made from DNA in aqueous solution and DNA-cetyltrimethylammonium chloride (CTMA) in an organic solvent. Z-scan measurements revealed a high third-order nonlinearity with n_2_ exceeding 10^−9^ at a wavelength of 1570 nm, for a nonlinarity about five orders of magnitude larger than that of silica. We also demonstrated ultrafast saturable absorption (SA) with a modulation depth of 0.43%. DNA thin solid films were successfully deposited on a side-polished optical fiber, providing an efficient evanescent wave interaction. We built an organic-inorganic hybrid all-fiber ring laser using DNA film as an ultrafast SA and using Erbium-doped fiber as an efficient optical gain medium. Stable transform-limited femtosecond soliton pulses were generated with full width half maxima of 417 fs for DNA and 323 fs for DNA-CTMA thin-solid-film SAs. The average output power was 4.20 mW for DNA and 5.46 mW for DNA-CTMA. Detailed conditions for DNA solid film preparation, dispersion control in the laser cavity and subsequent characteristics of soliton pulses are discussed, to confirm unique nonlinear optical applications of DNA thin-solid-film.

Since the discovery of the double helix structure of deoxyribonucleic acid (DNA) in 1953^1^,^2^, DNA has been at the center of interdisciplinary scientific research. Along with ever-increasing biochemical applications, DNA has recently attracted keen attention from physical scientists as a new organic alternatives to current inorganic functional materials for electronics and optoelectronics applications^3^,^4^,^5^,^6^,^7^,^8^,^9^,^10^,^11^,^12^,^13^. The unique promise of DNA as a photonic material was first recognized by the use of DNA as a bulk host to an optical dye, demonstrating a significant optical gain in the visible range^14^,^15^. In form of nano-crystalline layers, form, DNA has also shown high potential as an electron-blocking layer in organic light-emitting diodes (OLED), producing noticeable luminosity increases by large factors^16^,^17^,^18^. In an optical waveguide structure, DNA solid film has been employed as a cladding layer for polymeric electro-optic modulators^19^, and all-DNA electro-optic waveguide modulators have also been demonstrated^20^. However, these prior attempts have used the electro-optic and optical properties of DNA only in a linear optics regime at a moderate switching frequency.

A complex nonlinear refractive index of DNA in aqueous solution has been reported by Samoc *et al*.^21^, using the Z-scan method. DNA has been used as a dopant in a polyvinyl alcohol (PVA) host with a concentration of less than 1.5 wt.%, to modify the nonlinear optical response of PVA film^22^. Recently DNA has also been used as a polymeric host for graphene nano-flakes^23^ and nonlinear optical dyes^24^, where their nonlinear optical properties have mainly been attributed to the dopants rather than to the DNA host. Thus far systematic analyses on the nonlinear optical response of DNA solid film itself as a photonics material have not yet been reported; neither has the realization of a DNA-based nonlinear optical device in an ultrafast laser system been attempted.

In the case of DNA, the most abundant biopolymer, nonlinear refractive indices of DNA in water solution were reported^21^ but the measurements revealed only a moderate nonlinearity with n_2_ in the range of 10^−15^~10^−14^ cm^2^/W, which is of the same order as n_2_ in pure water, or 0.5~1 × 10^−15^ 21. The DNA concentration in the aqueous solution was a relatively low 0.0837 wt% 21 and in a higher DNA concentration, the solution became significantly turbid with very high scattering losses, which make it possible to use as an optical medium, especially in the nonlinear regime. In our study, we successfully solidified DNA solutions into an optically transparent thin solid film, achieving an increase in the optical nonlinear index n_2_ of more than three orders of magnitude compared with prior DNA aqueous solutions^21^, as well as higher optical transmission over a very broad spectral range from the visible to the infrared.

Saturable absorbers (SAs) have been one key type of nonlinear optical devices, and are being used for both passive mode-locking and Q-switching of lasers to generate short optical pulse trains^25^. Ultrafast pulsed lasers have found a wide range of applications, including materials processing^26^, optical communications^27^, and biomedical instrumentation^28^. Conventional bulk SAs have been realized in various forms, such as organic dyes^29^,^30^, color filter glasses^31^, and ion-doped crystals^32^. However, prior SAs have shown their own fundamental limitations in terms of optical damage threshold^33^, response time^33^, spectral range of operation^34^, and seamless integration into existing compact solid state laser cavities^33^. As an alternative to these bulk SAs, semiconductor saturable absorber mirrors (SESAMs) have been developed using quantum well technologies^35^. SESAMs offer a very small form factor and high efficiency but their spectral response range adjustments require a highly sophisticated band gap engineering and fabrication process. In recent years, SAs have been demonstrated using not only carbon nano-materials such as carbon nanotubes (CNTs) and graphene, but also using nano-sheet materials including MoS_2_ and WS_2_ in fiber laser cavities^36^,^37^,^38^,^39^,^40^,^41^. There was extensive investigation during the last two years based on nano-sheet materials, following the first innovating implementation of MoS_2_ as a saturable absorber in ultrafast photonics^37^. Moreover, black phosphorous has recently attracted attention as a natural candidate for broadband optical applications such as ultra-fast photonics devices, and it can be considered as a biocompatible material, as it can degrade in human blood due to the acidic environment^42^. These nano-material based SAs provide a wide spectral range of operation, along with high integration capability. However, there have been increasing concerns that these nano-materials might affect human health after long term exposure^43^. Therefore a bio-compatible alternative would be highly desirable for developing environmentally-friendly and non-hazardous photonic devices that could find rapidly increasing applications in *in-vivo* or *in-situ* biomedical laser processing and sensing. A few photonic applications have been demonstrated in recent years using bio-compatible materials, such as single-cell biological lasers^44^, optofluidic biolasers^45^, and silk fibroin lasers^46^. These efforts in bio-compatible photonics have been confined only to lasers and light emitting devices in relatively low power domain and in the linear regimes. Nonlinear optics has recently been applied to biomaterials for various applications such as high resolution imaging^47^, supercontinum generation^48^, and an alternative nonlinear optical material^49^, to name a few. However, one important application of nonlinear optics, ultrafast pulse generation in fiber laser cavities, has not yet been fully explored in biocompatible materials including DNA, despite its high importance and potential.

In the present study, the authors report a new side of DNA’s physical properties, unique nonlinear optical behavior of DNA thin solid film in the femtosecond regime, and the application of DNA as a biocompatible SA to realize an ultrafast Er-doped fiber laser, for the first time to the best knowledge of the authors. This research explores the potential of DNA as a functional photonic material to form a novel biocompatible hybrid photonic device. In this investigation, we focused on two types of DNA thin solid films made from: 1) DNA dissolved in water and 2) DNA-cetyltrimethylammonium chloride (CTMA) in an organic solvent, without any optically functional additives, in order to quantify the optical nonlinearity of DNA solid thin film itself, for the first time. Using a well-established femtosecond Z-scan technique^50^, we experimentally quantified the nonlinear absorption coefficient, nonlinear refractive index, and third-order nonlinear susceptibility of DNA and DNA-CTMA solid thin films at 800 and 1570 nm. We further investigated intensity-dependent optical transmission to observe saturable absorption and ultrafast pump-probe spectroscopy to confirm the potential of DNA as an efficient SA. Note that prior dye lasers used an organic gain medium and an organic saturable absorber in the visible range^51^ as in Fig. 1a, while conventional solid state lasers consisted of inorganic counterparts^52^ as in Fig. 1b. In this study, we combined a DNA-based organic SA with an Erbium doped fiber as an inorganic gain medium in a compact all-fiber ring cavity as shown in Fig. 1c, and we successfully mode-locked a ring cavity to generate stable transform-limited femtosecond soliton pulse trains in the anomalous dispersion regime at an eye-safe wavelength of 1.5 μm, for the first time.

## Materials and Methods

**Figure 2:**
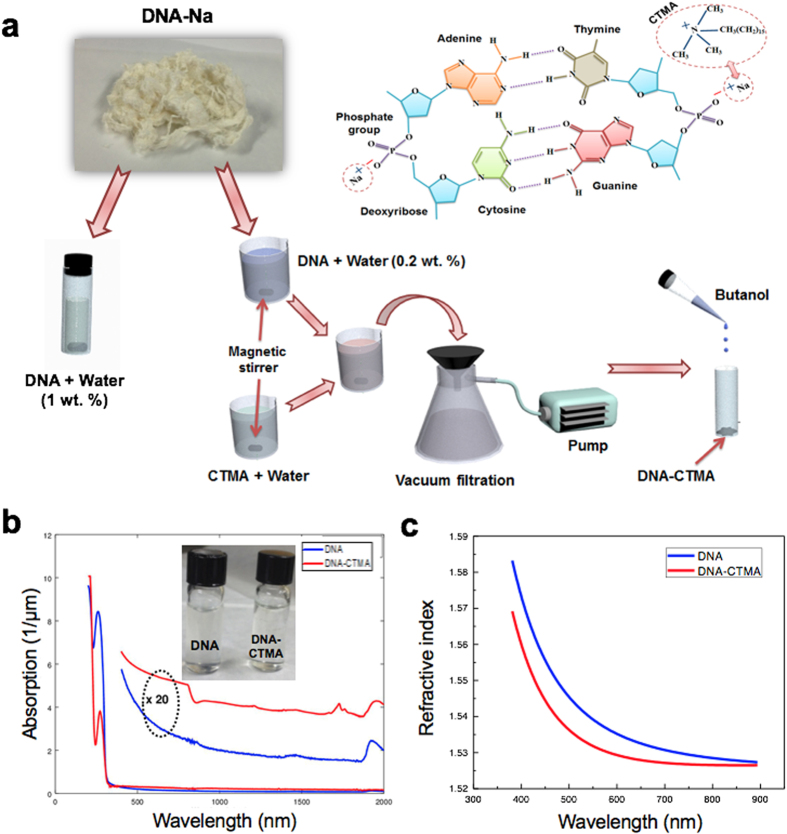
(**a**) Schematic representation of the DNA molecule and its preparation process for the aqueous DNA solution and DNA-CTMA dissolved in butanol. (**b**) Absorption spectra of the spin-coated solutions on the quartz substrate, (inset: the prepared DNA solutions). (**c**) Refractive index of the two thin films, measured by an ellipsometer.

The double-helix structure of DNA consists of two linear strands wound around each other^1^,^2^, as shown in Fig. 2a. This double helical structure with π electron-rich base pair stacking is preserved in both the “wet” and “dry” forms^53^. DNA solid films have been deposited on various substrates by slowly evaporating a relatively high concentration DNA-water solution, and their linear refractive index dispersions have been reported in the UV-visible-near IR region^54^. In order to further extend well-established thin film techniques, a chemical complex composed of DNA and cetyltrimethylammonium chloride (CTMA) has been used in organic solvents for spin coating processes^15^. DNA-CTMA films have shown potential for photonics and optoelectronics applications in the linear and relatively low-intensity optical regimes^55^,^56^.

In this research, we used B-type DNA processed from salmon roe^14^,^15^, which had an average molecular weight of >8 MDa, or a fragment size of longer than 40 microns. DNA-based solutions were prepared similar to a prior procedure^54^,^56^, as depicted in Fig. 2a. Purified DNA derived from salmon was filtered and mixed into deionized water. The 1 wt.% DNA aqueous solution was used to form pure DNA thin solid films by a slow evaporation technique^54^. The 0.2 wt.% DNA aqueous solution was further complexed into an aqueous solution of CTMA to form DNA-CTMA precipitates^56^. Dried DNA-CTMA precipitates were mixed into a 1 wt.% butanol solution. The inset of Fig. 2b shows the colorless aqueous solution of DNA and DNA-CTMA dissolved in butanol. These solutions were spin-coated onto plasma treated quartz substrates to form thin solid DNA films, whose thicknesses were 710 nm and 145 nm for DNA and DNA-CTMA, respectively. All the DNA films were vacuum dried at an elevated temperature for 24 hours before optical measurements in order to minimize the impact of humidity^54^. Optical transmission through the films was measured from the UV to the IR and the results are summarized in Fig. 2b. The characteristic 260 nm absorption peak of DNA was clearly identified in both films, and corresponds to the electronic transitions of the four heterocyclic bases in the nucleic acids^57^,^58^. We found the DNA film slowly evaporated from 1 wt.% water solution did have a stronger absorption peak than DNA CTMA film which started from 0.2 wt.% water solution. We also fabricated DNA thin solid films on silicon wafer substrates with almost the same thicknesses to measure the linear refractive indices using an ellipsometer, and the results are summarized in Fig. 2c. DNA film prepared from aqueous solution showed a higher refractive index than DNA-CTMA film, consistent with prior reports^54^,^55^. Knowledge of these linear refractive indices, *n*_*0*_*(λ)*, is an essential prerequisite to further characterize the nonlinear optical characteristics of DNA in terms of *n*_*2*_ and *χ*^*3*^, and will be discussed in the following sections.

### Nonlinear optical properties characterized by femtosecond Z-scan measurements

In principle, one can measure intensity- or fluence-dependent nonlinear transmission change with an open-aperture Z-scan. However, this method has shown limitations in measurement precision, especially for nanomaterials whose spatial distribution varies within a sample. The incident beam diameter substantially changes to vary the light fluences, and consequently the non-uniform sample area within the beam diameter can result in measurement errors. To overcome this problem, we developed a nonlinear transmission measurement setup with the high resolution of about 0.1% to changes in transmission intensity, while the incident beam diameter is kept constant. This method has been successfully implemented to the characterization of nonlinear optical properties of nano-materials whose surface morphology were not sufficiently homogeneous^59^, and we also implemented this technique in our DNA thin solid films in order to enhance the measurement precision.

**Figure 3:**
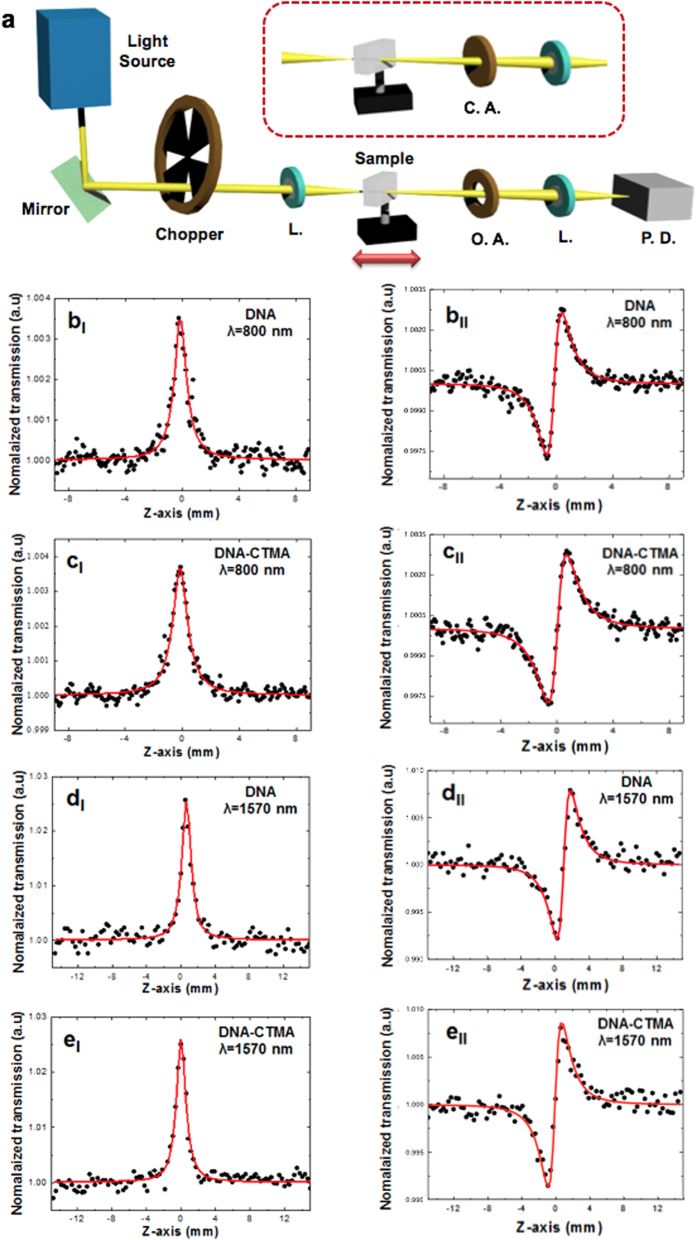
(**a**) Experimental set-up for Z-scan measurements (L: Lens, O. A.: Open aperture, C. A.: Closed aperture, P. D.: Photodetector). (**b**)**–**(**e**) Measured Z-scan data overlaid with theoretical fittings. The top four figures show measurements taken at λ = 800 nm, while the bottom four figures show measurements at λ = 1570 nm. The figures in the left column are open-aperture Z-scan data, while the right column shows closed-aperture Z-scans.

Nonlinear optical properties of Salmon DNA in aqueous solution have been measured using a similar Z-scan technique^21^. However, the DNA concentration was a relatively low 0.0837 wt% 21 and the measurements showed only a moderate nonlinearity with *n*_*2*_ in the range of 10^−15^ ~ 10^−14^ cm^2^/W, which is the same order as *n*_*2*_ of pure water 0.5 ~ 1 × 10^−15^. In this paper, the nonlinear refractive index of solid thin film of DNA and DNA-CTMA were fully investigated for the first time, which revealed the unexpectedly efficient and versatile nonlinear optic nature of DNA solid thin film. We used an accurate single-beam Z-scan technique to characterize the nonlinear refractive index (*n*_*2*_) and nonlinear absorption coefficient (*β*) of DNA and DNA-CTMA thin solid films, and data were analyzed following prior reports^50^. We used a Ti:sapphire laser operating at λ = 800 nm with a pulse duration of 110 fs, repetition rate of 79.8 MHz and an average power of 172 mW. The laser was focused to a beam waist of 12.83 μm with an intensity of 6.69 GW/cm^2^. In addition, a synchronously pumped optical parametric oscillator (SPOPO) was used for the measurements at λ = 1570 nm with a pulse duration of 110 fs, repetition rate of 79.8 MHz and an average power of 17 mW. The laser was focused to a beam waist of 5.17 μm with an intensity of 4.06 GW/cm^2^. For the Z-scan measurements at both wavelengths, the peak intensity at the focus was kept at a similar level for better comparison. After focusing the laser beam, the DNA thin solid film samples were then translated near the focal point using a motorized stage. The transmitted laser beam power was measured through either an open aperture (OA) or a closed aperture (CA) by a single photo-detector. A schematic diagram of the Z-scan measurements is shown in Fig. 3a. Here we used the prepared DNA and DNA-CTMA thin solid films on quartz substrates with thicknesses of 710 nm and 145 nm, respectively.

In OA Z-scan, we measured the transmission ratio (*Δψ*_*o*_), which is the ratio between the transmission at an axial position z near the focal point and that of the far field. In CA Z-scan measurements, we measured the phase shift (*ΔФ*_*o*_) by translating the sample. From *Δψ*_*o*_ and *ΔФ*_*o*_ we calculated the nonlinear constants: the nonlinear absorption coefficient 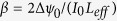, the nonlinear refractive index coefficient 
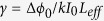
, and the real and imaginary parts of the third order nonlinear susceptibility, 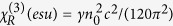 and 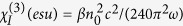. Here *L*_*eff*_ *=* (*1* − *e*^*−αL*^)/*α*, with the sample thickness *L* and the linear absorption coefficient *α* shown in Fig. 2b. *I*_*0*_*, c*, and *n*_*0*_ are the on-axis irradiance at the focal point, speed of light in vacuum, and the linear refractive index as shown in Fig. 2c, respectively. To eliminate contributions of the quartz substrate to the nonlinear optical response, we measured a pristine quartz substrate and we confirmed these nonlinear responses were solely attributable to the DNA thin solid films.

OA and CA Z-scan traces are summarized in Fig. 3b–e along with theoretical fittings^50^. Nonlinear optical constants of DNA thin solid films are presented in Table 1, along with corresponding measurements in DNA-water solution^21^. In prior DNA aqueous solution^21^ laser-induced micro-bubbles in water resulted in nonlinear scattering, which inevitably increased experimental errors and uncertainty in the measurements. In our study, we optimized the spin-coating and drying processes to fabricate optically transparent thin solid DNA films, which ensured a high measurement repeatability, and consistently low experimental errors, which are comparable to those of conventional solid film samples. The DNA and DNA-CTMA thin solid films showed nonlinear refractive index (*n*_*2*_) and nonlinear absorption coefficient (*β*) several orders of magnitude larger than those of the low concentration DNA in water solutions. In contrast to prior DNA aqueous solutions^21^, both *n*_*2*_ and *β* of DNA thin solid films decreased as the laser frequency increased. In comparison with the prior report, few-layer graphene showed a nonlinear refractive index of 10^−7^ cm^2^ W^−1^, which was 9 orders of magnitude larger than that of the bulk dielectric^60^, DNA thin film also needs to be optimized and studied in more depth.

### Saturable absorption measurements

**Figure 4:**
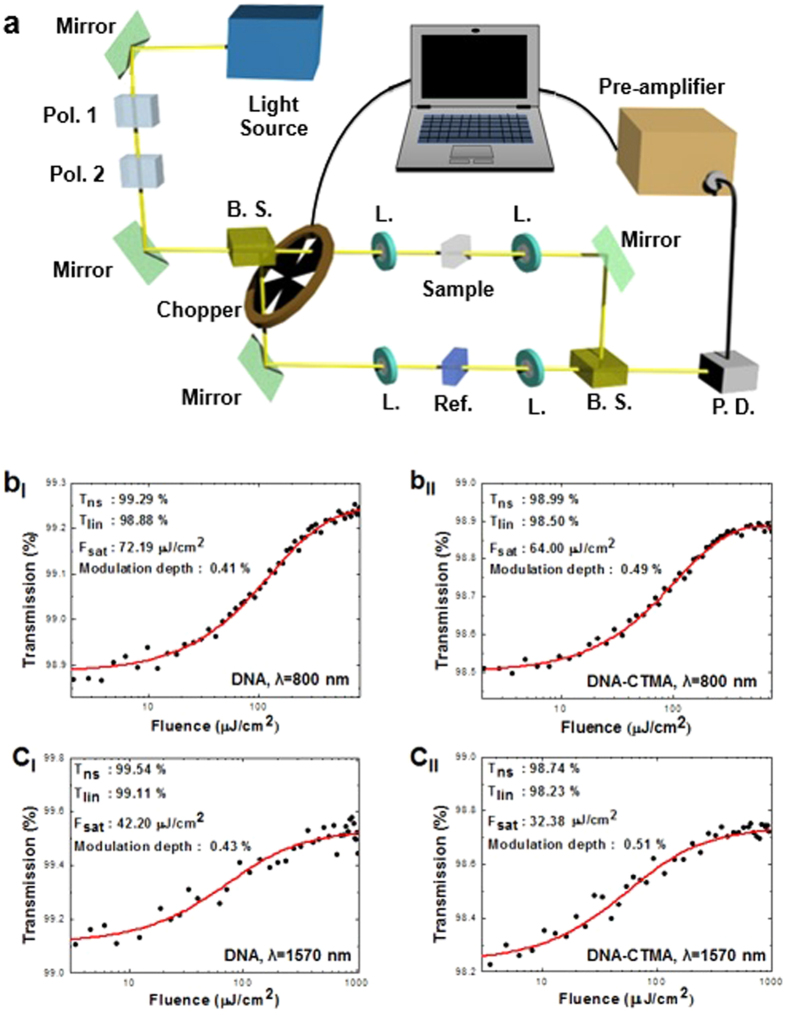
(**a**) Schematic of the nonlinear transmission measurement setup (Pol.: Polarizer, B. S.: Beam splitter, L.: Lens, P. D.: Photodetector). **(b)–**(**c**) Nonlinear transmission measurement results and fits for DNA and DNA-CTMA at 800 and 1570 nm, as labeled (T_ns_: Non-saturable transmission, T_lin_: Linear transmission, F_sat_: Saturation fluence).

We further pursued intensity-dependent nonlinear transmission measurements using the same lasers from the Z-scan, as shown in Fig. 4a. The measurements were taken from the DNA and DNA-CTMA thin films on quartz substrates whose physical parameters were identical to those used in the Z-scan experiments.

In the nonlinear transmission experiment, the fluence of the incident beam was controlled by crossing two polarizers and the transmission change through the DNA films was measured by photo detector. The laser beam was divided into two equal paths using a 50:50 beam splitter, the reference quartz substrate was mounted on one path, and the DNA-deposited quartz substrate on the other to subtract the substrate contributions. In order to achieve data with a high resolution of less than 0.1% for changes in transmission, the configuration of both paths was kept the same as the transmitted beam was measured with one photo detector. We used an electrical chopper as a high-speed optical shutter and measured transmissions through the reference and sample paths. The intensity-dependent transmission measurements are summarized in Fig. 4b and c at λ = 800 nm and 1570 nm, respectively, and experimental data were fitted to a two-level saturable absorber model^56^. We experimentally obtained the modulation depth, nonlinear saturable loss, and saturation fluence for DNA and DNA-CTMA thin solid films and the results are summarized in Table 2. Note that the magnitude of the modulation depth of DNA films was similar to that of Graphene layers first reported by Bao *et al*.^39^. The modulation depth can be further increased by optimization of DNA thin film processing technologies with an optimal DNA concentration, silica-DNA surface adhesion and the effective light-DNA interaction length, which are being pursued by the authors. We did not observe significant signs of any roll-over in the transmission, even at the maximum available pump power. Moreover, very low nonlinear saturable loss confirmed the high potential of DNA thin solid films as saturable absorbers for high peak power mode-locked lasers. Note that these nonlinear optical properties were directly obtained from DNA thin solid film without any functional additives, and this is the first report to quantify the nonlinear properties of pristine DNA thin solid films.

### All fiber DNA saturable absorber for mode-locking of an Erbium-doped fiber ring laser

The Z-scan and nonlinear transmission measurements of DNA thin solid films revealed a unique combination of a low non-saturable loss and a sufficiently high modulation depth, which are especially well-suited for a saturable absorber (SA) enabling a stable passive mode-locking without Q-switching instabilities^61^. In this report, we focused on the SA application of DNA thin solid film to realize femtosecond mode-locking of a fiber laser cavity combining optical gain from inorganic Er ions in silica glass and saturable absorption from DNA thin solid films, for the first time to the best knowledge of the authors.

**Figure 5:**
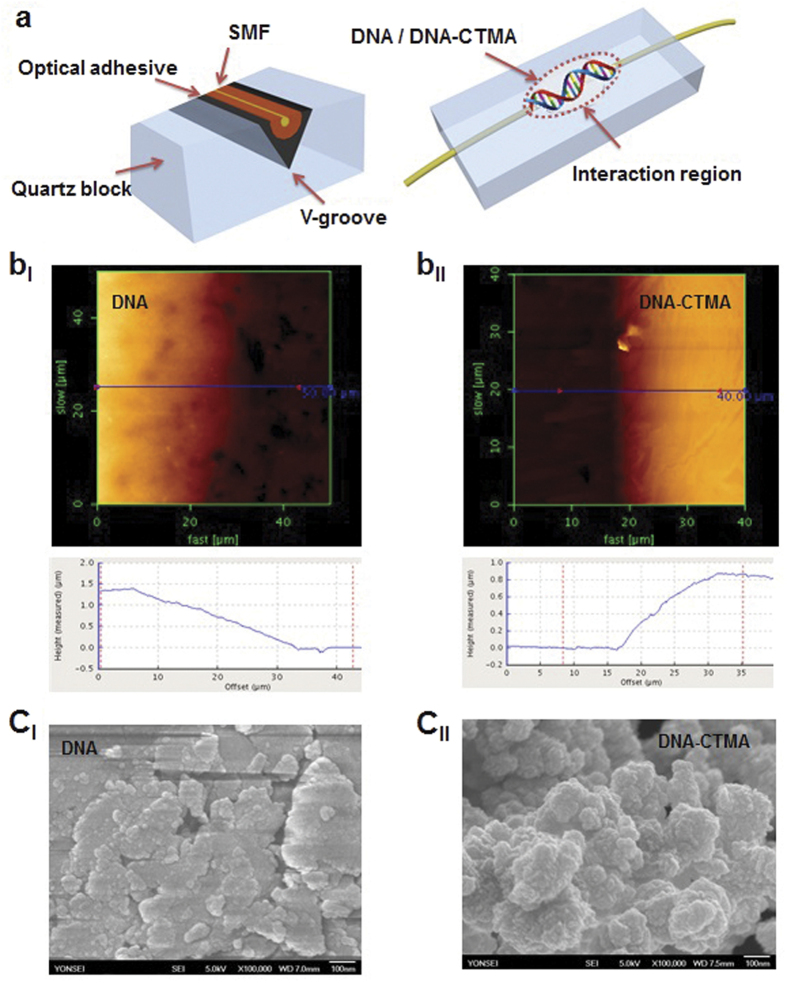
(**a**) Cross sectional and skew schematics of the side polished fiber (SPF) and its light interaction region. (**b**) (top) AFM morphologies of the DNA and DNA-CTMA thin films on SPF, and (below) their height profiles. (**c**) SEM morphologies of the DNA and DNA-CTMA thin films on SPF.

We deposited DNA and DNA-CTMA thin solid film on a side-polished fiber (SPF) to make an all-fiber DNA-based SA as shown in Fig. 5a. SPF can provide an efficient evanescent wave interaction (EWI) between the overlaid material and the propagating light. SPF-type SA can provide a longer optical interaction length, a higher optical damage threshold, and lower insertion loss than ferrule-type SAs^62^. The SPF was fabricated by polishing down the cladding of a buried single mode optical fiber in a V-groove silica block. DNA-aqueous solution and DNA-CTMA-butanol solution were dropped on the polished surface of SPF and then spin coated with optimal conditions, to make an all-fiber SA. The nonlinear optical response of the DNA-integrated fiber devices has been investigated and included in Supplemetary Information. The average thickness of the deposited film was measured to be ~1.43 μm for DNA and 0.87 μm for DNA-CTMA, as shown in Fig. 5bI and bII. Spin coated DNA-CTMA thin film formed more uniform layers than DNA thin film, due to the higher evaporation rate of butanol. Figure 5cI and cII show the field emission scanning electron microscopy (SEM) images of the deposited DNA thin films on SPF. Films showed a homogeneous distribution of surface morphology without any observable grain boundaries. The insertion loss of the pristine SPF was ~0.1 dB before the thin film deposition process. After deposition, DNA film SPF-SA had an insertion loss (IL) of ~5.5 dB, and a polarization dependent loss (PDL) of ~6.4 dB. DNA-CTMA film SFP-SA had an IL of ~4.3 dB and a PDL of ~3.9 dB. We could not measure any noticeable changes in these physical parameters as we increased the incident pump laser power. No pulse generation was observable for the blank SPF without DNA films, which confirmed that the ultrafast optical nonlinearity in the laser cavity did originate directly from the DNA thin solid film, and originated neither from nonlinear polarization rotation nor from nonlinear dispersion of the cavity.

**Figure 6:**
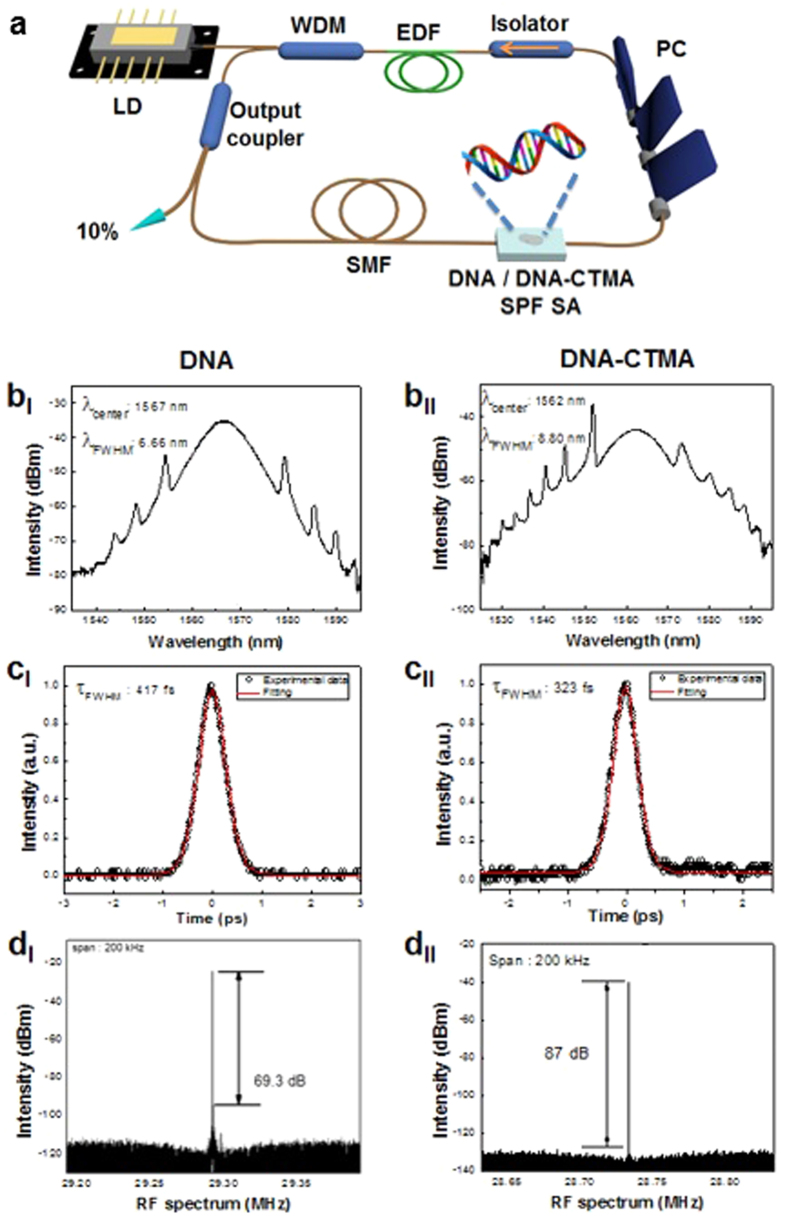
(**a**) Schematic diagram of the proposed all-fiber ring laser cavity using Erbium-doped fiber (EDF) as an optical gain medium and DNA film on side polished fiber as a SA. **(b)** Mode-locked soliton optical spectra (**c**) Autocorrelation traces of laser output. (**d**) Fundamental frequency of RF spectral profiles.

We constructed two ring cavities using DNA and DNA-CTMA SPF-SA, as schematically shown in Fig. 6a. One meter of erbium-doped optical fiber (ER80-8/125) was used as a gain medium common to both lasers. The total length of the ring resonator with the DNA SPF-SA was approximately 7.06 m and the total net cavity dispersion was adjusted to be −1.134 ps^2^ at λ = 1550 nm. The cavity with the DNA-CTMA SPF-SA had a length of 6.65 m with a net dispersion of −1.137 ps^2^. The ring cavities were pumped by a laser diode (LD) at λ = 976 nm through a 980/1550 nm wavelength division multiplexing (WDM) coupler. A polarization controller (PC) was inserted to control the polarization state inside the laser cavity. A polarization-insensitive optical isolator was used to eliminate back-reflection and to maintain the unidirectional light propagation. A directional tap coupler with an output coupling ratio of 10% was inserted to monitor the spectral and temporal characteristics of the laser using an optical spectrum analyzer (Yokogawa AQ6370 B), an autocorrelator (Femtochrome; FR-103HS), a digital oscilloscope (Tektronix TDS 784D), and a radio-frequency (RF) spectrum analyzer (Agilent technologies N9000A).

Self-started ultrafast pulses were successfully generated and the output characteristics of the fiber lasers are summarized in Fig. 6b–d. In the optical spectral domain, typical soliton-like pulse shapes with characteristic Kelly-bandside peaks were obtained for the cavity with DNA SPF-SA at a pump LD power of 169 mW and for the cavity with DNA-CTMA SPF-SA at 238 mW, as shown in Fig. 6bI and bII, respectively. The spectra had their center wavelengths at λ = 1567 nm with a 3-dB bandwidth of 6.66 nm for the DNA SPF-SA cavity, and at λ = 1562 nm with a bandwidth of 8.80 nm for the DNA-CTMA SPF-SA cavity. The repetition rates of the pulse trains were measured using both an oscilloscope and an RF spectrum analyzer, and the results are summarized in Supplementary Figure 2. The pulse repetition rate was 29.29 MHz for the DNA and 28.73 MHz for DNA-CTMA SPF-SA cavity, which corresponded well to their cavity lengths. In the temporal domain, the measured pulse trace was well-fitted with a Sech^2^-profile and their full width half maximums (FWHMs) were 417 fs for the DNA SPF-SA cavity and 323 fs for the DNA-CTMA SPF-SA cavity, as shown in Fig. 6CI and 6CII, respectively. In the frequency domain, the output pulse showed a very high signal-to-noise ratio of 69.3 dB for the DNA cavity and 87 dB for the DNA-CTMA cavity, as shown in Fig. 6dI and dII. The average output power of the cavities was 4.20 mW for the DNA cavity and 5.46 mW for the DNA-CTMA cavity, which can be improved with further optimization of the optical gain and the output coupling ratio. We could not observe any optical damage such as melting or fragmentation of DNA films, even at the highest available LD pump power.

All the measurements in Fig. 6 confirmed highly stable operation of the mode-locked lasers to generate soliton pulse trains without Q-switching instabilities. The differences between the two types of lasers might have originated from how the DNA solution wetted the SPF surface in the spin-coating process, and how the liquid film was solidified into a thin solid film. DNA-CTMA films were fabricated from DNA-CTMA dissolved in butanol solvent. Due to its low viscosity Butanol wets the surface of the side-polished fiber block substrate sufficiently well, and it evaporates much faster than water, which might have provided a more uniform film deposition than DNA-water solution.

## Results and Disscusion

High optical nonlinearity of DNA film in the infrared region, as summarized in Tables 1 and 2 was a rather unexpected discovery, because DNA only has a distinctive UV absorption band^63^ near λ = 260 nm, as shown in Fig. 2b, and there are no direct optical transitions in DNA either at λ = 800 nm or λ = 1570 nm. The intensity-dependent optical nonlinearity of a material has been attributed to the saturation of the material’s absorption band^61^. Saturable absorbers have been phenomenologically modeled as a nonlinear optical process between a pair of two specific electronic states, a ground state and an excited state, whose energy difference matches the incident photon energy^64^. We adopted this qualitative model to explain the nonlinear optical process in DNA thin solid film.

DNA is a bio-polymer and its optical absorption is affected by a complex configuration of the highest occupied molecular orbital (HOMO) and the lowest unoccupied molecular orbital (LUMO). The energy gap between HOMO and LUMO in an amorphous polymer can result in an extended absorption tail toward the longer wavelength, analogous to Urbach tail in disordered materials^65^. The Urbach absorption tail is a universal phenomenon in amorphous materials, and such spectral extension of the absorption band into the spectral region of wavelength longer than that of the band gap is known to be related with the defect states^66^,^67^,^68^,^69^,^70^,^71^. In recent studies on the photo-conductivity of DNA, water in DNA has been reported to generate temporal or structural defects, which led to an electronic contribution to low-frequency absorption^53^. In our film preparation as shown in Fig. 2a, the process inevitably required direct contact between water molecules and the DNA, and there could be various types of defects related with water and other ions, which might provide an extended absorption into the visible and IR regions. Owing to the amorphous and wide band-gap nature of DNA thin solid film, an extended absorption reaching to the near IR region was seen. We measured UV-VIS-IR transmission of a DNA solid film with an average thickness of 60 μm, and the absorption band extended from UV to IR range in a monotonically decreasing manner. This absorption tail might be analogous to the Urbach tail observed in amorphous materials^72^, although it may also be due to scattering losses observed at higher frequencies in DNA. Due to this wavelength dependent transmission, the nonlinearity also showed a spectral dependence at λ = 850 nm and 1550 nm. Further investigation of DNA’s optical nonlinearity in a wider spectral range could give more information on the origin of saturable absorption and is being pursued by the authors.

The optical nonlinearities of DNA films are compared with other organic chemicals and inorganic counterparts in Table 3. It is noteworthy that DNA showed nonlinearities comparable to prior organic SA chemicals and the initially reported modulation depth of Graphene^39^. Recently Graphene provided an order of magnitude larger modulation depth than our DNA thin solid films. However, DNA showed equivalent performance to Graphene in terms of SA performance in a fiber laser cavity to generate short pulse train, demonstrating stable mode-locking of soliton pulses with a duration of a few hundred femtoseconds. Furthermore, DNA demonstrates significant advantages over conventional SAs materials, such as carbon nano-materials and 2D nano-sheets. First, due to rapidly maturing DNA synthesis technologies, virtually unlimited variations and modifications can be made to DNA, including changes to the nucleobase sequences, the backbone, and the chain length as well as the functional dopants^73^. Further optimization of nonlinear and linear optical properties in DNA, therefore, can be efficiently achieved utilizing custom-made, mass-producible DNA technologies. Prior SA materials have been supplied mainly in a flake colloidal suspension, which requires complicated processes to provide optical quality thin films. CVD grown films are available for these materials, yet additional processes to remove the film from the substrate and transfer it on the target devices cannot be omitted. In comparison, DNA can be directly spin-coated on the device surface drastically reducing process requirements. Finally, DNA thin solid film is inherently biocompatible, which can allow new bio-integrable, environmentally-friendly biophotonic applications.

**Figure 7:**
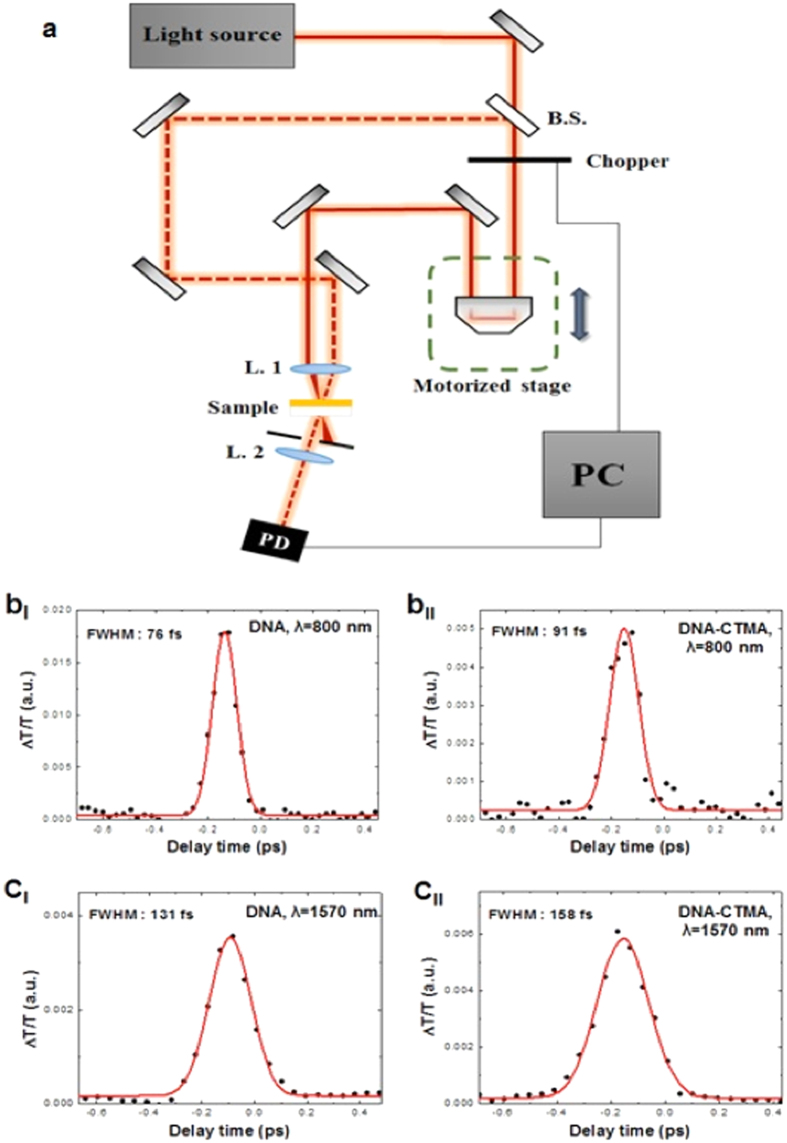
(**a**) Schematic of the Pump-probe measurement setup (B. S.: Beam splitter, L.: Lens, P. D.: Photodetector, PC: Personal computer). (**b,c**) Pump-probe measurement results and fits for DNA and DNA-CTMA thin solid film on quartz substrate at λ = 800 and 1570 nm, as labeled.

We further investigated the temporal response of solid DNA film by employing a time-resolved pump-probe spectroscopy set-up, which is similar to prior reports on two-dimensional nano-sheets^39^. The measurements were carried out using identical femtosecond lasers at λ = 800 and 1570 nm used in Z-scan experiments. Figure 7a schematically demonstrates the pump-probe measurement setup. The laser beam is divided at a 10:1 intensity ratio by a beam splitter and the two beams are focused on the sample by a plano-convex lens with a focal length of 7 cm. One beam is the pump-beam for exciting the sample and the other one is a probe-beam for observing carrier dynamics. The time delay between the pump- and probe-beams is controlled by a motorized stage in the path of the pump-beam. The results are summarized in Fig. 7b and c. It has been reported that the pump-probe experiments for graphene layers showed two decay components, the faster one with a decay time of ~90 femtoseconds and the slower one with a decay time of ~60 picoseconds^74^. In the case of DNA thin solid films, the temporal responses were well-fitted by exponential functions only with only a single fast decay component on the order of 100 femtoseconds. This fast decay component of DNA is comparable to those of prior 2D nano-sheets and is attributed to the stable mode locking capability of DNA saturable absorber, generating femtosecond pulses. The pump-probe results in Fig. 7 clearly show that the temporal overlap between two short pulses does exist in the IR spectral range within the DNA thin solid film, which is attributable to the ultrafast optical nonlinearity of DNA films. The observed properties can further open a new avenue of DNA solid thin film applications, such as broad spectral band mode-lockers, nonlinear optical switches, and optical limiters in combination with inorganic optical components to provide novel organic-inorganic hybrid photonic solutions.

## Conclusions

We have experimentally discovered that deoxyribonucleic acid (DNA) serves as a nonlinear optical material suitable for the development of hybrid organic-inorganic ultrafast fiber lasers. By measuring the linear and nonlinear optical transmission of DNA and DNA-CTMA thin solid films, high third order nonlinearity with nonlinear refractive index modulation depth sufficiently high for practical ultrafast saturable absorption were observed, which differs significantly from aqueous solution of DNA. Side-polished fibers (SPFs) were employed as a template to deposit DNA thin solid films which provided efficient evanescent wave interaction over a long distance. All-fiber ring laser cavities were fabricated including an erbium-doped optical fiber (EDF) gain medium and the DNA and DNA-CTMA films deposited on SPFs as saturable absorbers. Robust femtosecond soliton pulses were obtained with a pulse duration of 417 fs and 323 fs for DNA and DNA-CTMA SPF-SAs, respectively. Output pulses with very high signal-to-noise ratios over 69 dB were obtained using both DNA and DNA-CTMA SAs showing their capability to generate ultrafast pulse trains with high stability. The average output power was 4.20 mW for DNA and 5.46 mW for DNA-CTMA, but the power was limited only by the EDF optical gain and the output coupling ratio, which can be further optimized for higher output power. DNA thin solid films can open a new avenue of nonlinear optic device applications using biocompatible organic-inorganic hybridization.

## Additional Information

**How to cite this article:** Khazaeinezhad, R. *et al*. Ultrafast nonlinear optical properties of thin-solid DNA film and their application as a saturable absorber in femtosecond mode-locked fiber laser. *Sci. Rep.* **7**, 41480; doi: 10.1038/srep41480 (2017).

**Publisher's note:** Springer Nature remains neutral with regard to jurisdictional claims in published maps and institutional affiliations.

## Supplementary Material

Supplementary Information

## Figures and Tables

**Figure 1:**
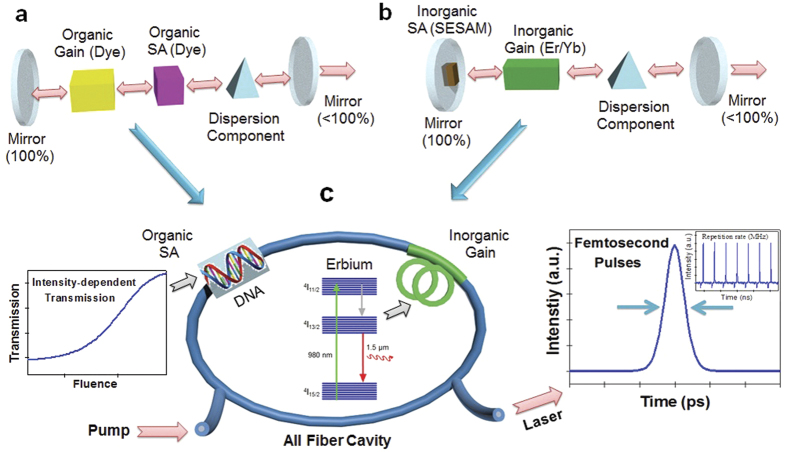
(**a**) Linear laser cavity including organic gain medium and organic saturable absorber (SA). (**b**) Laser cavity based on inorganic gain medium and inorganic SA. (**c**) Organic and inorganic hybrid all-fiber ring laser.

**Table 1:** Nonlinear absorption coefficient, nonlinear refractive index, and third order nonlinear susceptibility of DNA and DNA-CTMA thin solid films at 800 and 1570 nm.

Wavelength	Material	β (m/W)	n_2_ (cm^2^/W)	χ^3^ (esu)
**800** **nm**	DNA film	1.51 × 10^−10^	3.63 × 10^−13^	4.15 × 10^−11^
	DNA-CTMA film	7.71 × 10^−10^	1.81 × 10^−12^	2.05 × 10^−10^
**1570** **nm**	DNA film	1.78 × 10^−9^	3.44 × 10^−12^	8.42 × 10^−10^
	DNA-CTMA film	8.89 × 10^−9^	1.82 × 10^−11^	4.11 × 10^−9^
**530**~**1300** **nm** [ref. 21]	DNA in Water	2 × 10^−11^ (@530 nm)	1 × 10^−14^~2 × 10^−15^	—

The thin film thicknesses were 710 nm and 145 nm for DNA and DNA-CTMA, respectively.

**Table 2:** Nonlinear saturable loss and saturation fluence of DNA and DNA-CTMA thin solid film at λ = 800 and 1570 nm.

Wavelength	Material	Nonlinear saturable loss (%)	Saturation fluence(μJ/cm^2^)
**800** **nm**	DNA film	0.71	72.19
	DNA-CTMA film	1.01	64.00
**1570** **nm**	DNA film	0.46	42.20
	DNA-CTMA film	1.26	32.38

The thin film thicknesses were 710 nm and 145 nm for DNA and DNA-CTMA, respectively.

**Table 3:** Nonlinear optical properties of DNA, DNA-CTMA, organic chemical, and graphene.

SA Material	Modulation depth (%)	Saturation intensity (MW/cm^2^)	Pulse width
**DNA film** (@ 1.55 μm)	0.4	281	417 fs
**DNA-CTMA film** (@ 1.55 μm)	0.5	216	323 fs
**Organic Dye** (DODCI) (@ 530 nm)	1.3 ref. 75	125 ref. 76	10 ps ref. 75
**Graphene** ref. 39 (@ 1.55 μm)	6.2~66.5	0.61~0.71	756 fs

## References

[b1] Watson J. D. & Crick F. H. A Structure for Deoxyribose Nucleic Acid. Nature 171, 737–738 (1953). 13054692 10.1038/171737a0

[b2] Wilkins M. H. F., Stokes A. R. & Wilson H. R. Molecular Structure of Deoxypentose Nucleic Acids. Nature 171, 738–740 (1953). 13054693 10.1038/171738a0

[b3] Kwon Y. W., Choi D. H. & Jin J. I. Optical, electro-optic and optoelectronic properties of natural and chemically modified DNAs. Polym J 44, 1191–1208 (2012).

[b4] Steckl A. J. DNA – a new material for photonics? Nat Photonics 1, 3–5 (2007).

[b5] Cosnier S. & Mailley P. Recent advances in DNA sensors. ANLY 133, 984–991 (2008). 10.1039/b803083a 18645636

[b6] Kawabe Y., Wang L., Horinouchi S. & Ogata N. Amplified Spontaneous Emission from Fluorescent-Dye-Doped DNA–Surfactant Complex Films. Adv Mater 12, 1281–1283 (2008).

[b7] Houlton A., Pike A. R., Galindo M. A. & Horrocks B. R. DNA-based routes to semiconducting nanomaterials. Chem Commun 45, 1797–1806 (2009). 10.1039/b818456a 19319411

[b8] Kwon Y. W., Lee C. H., Choi D. H. & Jin J. I. Materials science of DNA. J Mater Chem 19, 1353–1380 (2009).

[b9] Mizoguchi K., Tanaka S., Ogawa T., Shiobara N. & Sakamoto H. Magnetic study of the electronic states of B-DNA and M-DNA doped with metal ions. Phys Rev B 72, 033106 (2005).

[b10] Yang D. . Novel DNA materials and their applications. Wiley Interdiscip Rev: Nanomed Nanobiotechnol 2, 648–669 (2010). 20730873 10.1002/wnan.111 PMC7169675

[b11] Singh T. B., Sariciftci N. S. & Grote J. G. Bio-Organic Optoelectronic Devices Using DNA. Adv Polym Sci 223, 189–212 (2010).

[b12] Singh B., Sariciftci N. C., Grote J. G. & Hopkins F. K. Bio-organic-semiconductor-field-effect-transistor based on deoxyribonucleic acid gate dielectric. J Appl Phys 100, 024514 (2006).

[b13] Kim S. H., Choi D. S. & Kim D. S. Single-molecule Detection of Fluorescence Resonance Energy Transfer Using Confocal Microscopy. J Opt Soc Korea 12, 107–111 (2008).

[b14] Kawabe Y., Wang L., Koyama T., Horinouchi S. & Ogata N. Light amplification in dye-doped DNA-surfactant complex films. Proc SPIE 4106, 369 (2000).

[b15] Wang L., Yoshida J., Ogata N., Sasaki S. & Kajiyama T. Self-Assembled Supramolecular Films Derived from Marine Deoxyribonucleic Acid (DNA)−Cationic Surfactant Complexes: Large-Scale Preparation and Optical and Thermal Properties. Chem Mater 13, 1273–1281 (2001).

[b16] Hagen J. A., Li W., Steckl A. J. & Grote J. G. Enhanced emission efficiency in organic light-emitting diodes using deoxyribonucleic acid complex as an electron blocking layer. Appl Phys Lett 88, 171109 (2006).

[b17] Sun Q. . Highly Efficient Quantum-Dot Light-Emitting Diodes with DNA−CTMA as a Combined Hole-Transporting and Electron-Blocking Layer. ACS Nano 3, 737–743 (2009). 19309174 10.1021/nn8009079

[b18] Chen I., Chiu Y., Fruk L. & Hung Y. Enhanced Light Emission from Blue Organic Light-emitting Devices with DNA Biopolymer. Quantum Electronics Conference & Lasers and Electro-Optics 568, 182–184 (2011).

[b19] Heckman E. M. . DNA biopolymer conductive cladding for polymer electro-optic waveguide modulators. Appl Phys Lett 98, 103304 (2011).

[b20] Heckman E. M., Yaney P. P., Grote J. G., Hopkins F. K. & Tomczak M. M. Development of an all-DNA-surfactant electro-optic modulator. Proc SPIE 6117, 61170K (2006).

[b21] Samoc M., Samoc A. & Grote J. G. Complex nonlinear refractive index of DNA. Chem Phys Lett 431, 132–134 (2006).

[b22] Nithyaja B., Misha H., Radhakrishnan P. & Nampoori V. P. N. Effect of Deoxyribonucleic Acid on Nonlinear Optical Properties of Rhodamine 6G-Polyvinyl Alcohol Solution. J Appl Phys 109, 023110–023113 (2011).

[b23] Husaini S., Lesko A., Heckman E. M. & Bedford R. G. Engineered bio-compatible graphene nanomaterials for nonlinear applications. Opt Mater Express 5, 102–112 (2015).

[b24] Sahraoui B., Pranaitis M., Gindre D., Niziol J. & Kazukauskas V. Opportunities of deoxyribonucleic acid complexes composites for nonlinear optical applications. J Appl Phys 110, 083117 (2011).

[b25] Keller U. Recent developments in compact ultrafast lasers. Nature 424, 831–838 (2003). 12917697 10.1038/nature01938

[b26] Fermann M. E., Galvanauskas A. & Sucha G. Ultrafast Lasers: Technology and Applications. (Marcel Dekker, 2003).

[b27] Rulliere C. Femtosecond Laser Pulses: Principles and Experiments. (Springer, 2005).

[b28] Dausinger F., Lichtner F. & Lubatschowski H. Femtosecond Technology for Technical and Medical Applications. (Springer, 2004).

[b29] Sarukura N., Ishida Y., Nakano H. & Yamamoto Y. cw passive mode locking of a Ti:sapphire laser. Appl Phys Lett 56, 814 (1990).

[b30] Penzkofer A. & Baumler W. Saturable absorption dynamics of DODCI. Opt Quant Electron 23, 439–459 (1991).

[b31] Snitzer E. & Woodcock R. 9C8 Saturable absorption of color centers in Nd3 + and Nd3 + Yb3 + laser glass. IEEE J Quant Electron 2, 627–632 (1966).

[b32] Zolotovskaya S. A. . Nd:KGd(WO4)2 laser at 1.35 mm passively Q-switched with V3+:YAG crystal and PbS-doped glass. Opt Mater 28, 919–924 (2006).

[b33] Keller U. Ultrafast Solid-State Lasers. (Elsevier, 2004).

[b34] Khazaeinezhad R. . Passive Q-Switching of an All-Fiber Laser Using WS 2-Deposited Optical Fiber Taper. IEEE Photon J 7, 1–7 (2015).

[b35] Keller U. Ultrafast solid-state laser oscillators: a success story for the last 20 years with no end in sight. Appl Phys B 100, 15–28 (2010).

[b36] Okhotnikov O., Grudinin A. & Pessa M. Ultra-fast fibre laser systems based on SESAM technology: new horizons and applications. New J Phys 6, 177 (2004).

[b37] Zhang H. . Molybdenum disulfide (MoS2) as a broadband saturable absorber for ultra-fast photonics. Opt. Express 22, 7249–7260 (2014). 24664073 10.1364/OE.22.007249

[b38] Mao D. . WS2 mode-locked ultrafast fiber laser. Sci. Rep. 5, 7965 (2015). 25608729 10.1038/srep07965 PMC4302320

[b39] Hasan T., Sun Z., Wang F., Bonaccorso F., Tan P. H., Rozhin A. G. & Ferrari A. C. Nanotube–Polymer Composites for Ultrafast Photonics. Adv Mater 21, 3874–3899 (2009).

[b40] Set S. Y., Yaguchi H., Tanaka Y. & Jablonski M. Ultrafast fiber pulsed lasers incorporating carbon nanotubes. IEEE J. Sel. Topics Quantum Electron. 10, 137–146 (2004).

[b41] Khazaeinezhad R., Kassani S. H., Jeong H., Yeom D. I. & Oh K. Mode-locking of Er-doped fiber laser using a multilayer MoS_2_ thin film as a saturable absorber in both anomalous and normal dispersion regimes. Opt Express 22, 23732–23742 (2014). 25321840 10.1364/OE.22.023732

[b42] Lu S. B. . Broadband nonlinear optical response in multi-layer black phosphorus: an emerging infrared and mid-infrared optical material. Opt. Express 23, 11183–11194 (2015). 25969214 10.1364/OE.23.011183

[b43] Nel A., Xia T., Mädler L. & Li N. Toxic potential of materials at the nanolevel. Science 311, 622–627 (2006). 16456071 10.1126/science.1114397

[b44] Gather M. C. & Yun S. H. Single-cell biological lasers. Nat Photonics 5, 406–410 (2011).

[b45] Fan X. & Yun S. H. The potential of optofluidic biolasers. Nat Methods 11, 141–147 (2014). 24481219 10.1038/nmeth.2805 PMC4162132

[b46] Toffanin S. . Low-threshold blue lasing from silk fibroin thin films. Appl Phys Lett 101, 091110 (2012).

[b47] Bouma B. E., Tearney G. J., Compton C. C. & Nishioka N. S. High-resolution imaging of the human esophagus and stomach *in vivo* using optical coherence tomography, Gastrointestinal Endoscopy 51, 467–474 (2000). 10744824 10.1016/s0016-5107(00)70449-4

[b48] Cho Y. . Broadband supercontinuum generation using a hollow optical fiber filled with copper-ion-modified DNA. Opt. Express 23, 13537–13544 (2015). 26074601 10.1364/OE.23.013537

[b49] Zongo S. . Nonlinear optical properties of natural laccaic acid dye studied using Z-scan technique. Optical Materials 46, 270–275 (2015).

[b50] Sheik-Bahae M. Said A. A., Wei T. H., Hagan D. J. & Van Stryland E. W. Sensitive measurement of optical nonlinearities using a single beam. IEEE J Quant Electron 26, 760–769 (1990).

[b51] Wittman M., Penzkofer A. & Baumler W. Generation of frequency tunable femtosecond pulses in a cw pumped linear dispersion-balanced passive mode-locked rhodamine 6G dye laser. Opt Commun 90, 182–192 (1992).

[b52] Keller U. . Semiconductor saturable absorber mirrors (SESAM’s) for femtosecond to nanosecond pulse generation in solid-state lasers. IEEE J Sel Top Quantum Electron 2, 435–453 (1996).

[b53] Hübsch A., Endres R. G., Cox D. L. & Singh R. R. P. Optical conductivity of Wet DNA. Phys Rev Lett 94, 178102 (2005). 15904338 10.1103/PhysRevLett.94.178102

[b54] Samoc A., Miniewicz A. & Samoc M. & Grote, J. G. Refractive-Index Anisotropy and Optical Dispersion in Films of Deoxyribonucleic Acid. J Appl Polym Sci 105, 236–245 (2007).

[b55] Grote J. G. . DNA-based materials for electro-optic applications: current status. Proc SPIE 5934, 593406 (2005).

[b56] Heckman E. M., Hagen J. A., Yaney P. P., Grote J. G. & Hopkins F. K. Processing techniques for deoxyribonucleic acid: Biopolymer for photonics applications. Appl Phys Lett 87, 211115 (2005).

[b57] Manchester K. L. When are nucleic acids not nucleic acids? Problems with estimation of nucleic acid purity by UV absorbance. Biochem Educ 25, 214–215 (1997).

[b58] Omerzu A., Mihailovic D., Anzelak B. & Turel I. Optical spectra of wet and dry M-DNA. Phys Rev B 75, 121103 (2007).

[b59] Lee H. W. . Evaluation of the third-order optical nonlinearity of Au:SiO2 nanocomposites in the off-resonant spectral region. Opt. Commun. 286, 347–352 (2013).

[b60] Zhang H. . Z-scan measurement of the nonlinear refractive index of graphene. Opt. Lett. 37, 1856–1858 (2012). 22660052 10.1364/OL.37.001856

[b61] Haiml M., Grange R. & Keller U. Optical characterization of semiconductor saturable absorber. Appl Phys B 79, 337–339 (2004).

[b62] Bao Q. . Broadband graphene polarizer. Nat Photonics 5, 411–415 (2011).

[b63] Janiak C. A critical account on π–π stacking in metal complexes with aromatic nitrogen-containing ligands. J Chem Soc, Dalton Trans 2000, 3885–3896 (2000).

[b64] Garside B. K. & Lim T. K. Laser mode locking using saturable absorbers, J. Appl. Phys. 44, 2335 (1973).

[b65] Urbach F. The Long-Wavelength Edge of Photographic Sensitivity and of the Electronic Absorption of Solids. Phys Rev 92, 1324 (1953).

[b66] Wiley J. D., Thomas D., Schonherr E. & Breitschwerdt A. The absorption edges of GeS and Urbach’s rule. J Phys Chem Solids 41, 801–807 (1980).

[b67] Mohler E. & Thomas B. Experimental Test of Theoretical Models for Urbach’s Rule at Excitonic Absorption Edges. Phys Rev Lett 44, 543 (1980).

[b68] Cody G. D., Tiedje T., Abeles B., Brooks B. & Goldstein Y. Disorder and the Optical-Absorption Edge of Hydrogenated Amorphous Silicon. Phys Rev Lett 47, 1480 (1981).

[b69] Dunstan D. J. Evidence for a common origin of the Urbach tails in amorphous and crystalline semiconductors. J Phys C: Solid State Phys 15, L419 (1982).

[b70] Monroe D. & Kastner M. A. Exactly exponential band tail in a glassy semiconductor. Phys Rev B 33, 8881 (1986). 10.1103/physrevb.33.8881 9938315

[b71] Tamaoka J. & Komagata K. Determination of DNA base composition by reversed-phase high-performance liquid chromatography, Microbiol. Lett. 25, 125–128 (1984).

[b72] John S. Theory of Electron Band Tails and the Urbach Optical-Absorption Edge. Phys. Rev. Lett. 57, 1777 (1986). 10033542 10.1103/PhysRevLett.57.1777

[b73] Metzker M. L. Sequencing technologies-the next generation. Nature reviews genetics 11, 31–46 (2010). 10.1038/nrg2626 19997069

[b74] Jingzhi S. . Femtosecond UV-pump/visible-probe measurements of carrier dynamics in stacked graphene films. App. Phys. Lett. 97, 163103 (2010).

[b75] Burgess M. D. J., Fedosejevs R., Jaanimagi P. & Richardson M. C. Active-Passive Mode-Locked Flashlamp-Pumped Dye Laser. IEEE J Quant Electron 15, 1037–1038 (1979).

[b76] Zhu X. R. & Harris J. M. Influence of photoisomerization on saturated absorption of 3,3′-diethyloxadicarbocyanine iodide (DODCI) studied by diffraction from laser-induced, anharmonic thermal gratings. J Chem Phys 124, 321–332 (1988).

