# Supplementary material for: Ultrafast nonlinear optical properties of thin-solid DNA film and their application as a saturable absorber in femtosecond mode-locked fiber laser
Source: Sci Rep. 2017 Jan 27;7:41480. doi: 10.1038/srep41480 (PMC5269743; doi:10.1038/srep41480)
Supplement: Supplementary Information [file srep41480-s1.doc]

**SUPPLEMENTARY INFORMATION**

Ultrafast nonlinear optical properties of thin-solid DNA film and their application as a saturable absorber in femtosecond mode-locked fiber laser

Reza Khazaeinezhad^1,2,3^, Sahar Hosseinzadeh Kassani^1,2,3^, Bjorn Paulson^1^, Hwanseong Jeong^4^, Jiyoon Gwak^4^, Fabian Rotermund^4,5^*, Dong-Il Yeom^4^*, Kyunghwan Oh^1^*

^1^Photonic Device Physics Laboratory, Institute of Physics and Applied Physics, Yonsei University, Seoul 120–749, South Korea

^2^Harvard Medical School, Boston, Massachusetts 02115, USA

^3^Wellman Center for Photomedicine, Massachusetts General Hospital, Boston, Massachusetts 02114, USA

^4^Department of Physics & Energy Systems Research, Ajou University, Suwon 443-749, South Korea

^5^Department of Physics, Korea Advanced Institute of Science and Technology, Daejeon 34141, South Korea

*Corresponding authors

Kyunghwan Oh

Fax: +82-2-365-7657; Tel: +82-2-2123-5608; E-mail: koh@yonsei.ac.kr

Dong-Il Yeom

Fax: +82-31-219-1615; Tel: +82-31-219-2576; E-mail: diyeom@ajou.ac.kr

Fabian Rotermund

Fax: +82-42-350-2510; Tel: +82-42-350-2518; E-mail: rotermund@kaist.ac.kr

In order to characterize the nonlinear optical response of the fabricated DNA devices, we used the transmission measurement setup shown in Supplementary Figure 1 (a). A stable Er-doped optical fiber laser operates at a central wavelength of 1563 nm with pulse duration of 800 fs and a pulse repetition rate of 41 MHz. A computer-controlled motor-driven variable optical attenuator (VOA) adjusts the input optical power.

Supplementary Figure 1 (b) summarizes nonlinear transmission measurements and fittings for the DNA and DNA-CTMA thin solid films deposited on side polished fibers (SPFs). Intensity-dependent transmission was clearly observed and the transmission increased with the input laser peak intensity in a nonlinear manner. Hysteresis in transmission was not observed within the available laser power range. We carried out the same experiments for a blank SPF without DNA films, but the transmission stayed the same independent of the incident laser peak power, which confirmed that the observed saturable absorption characteristics are contributed only by the DNA thin films.

**
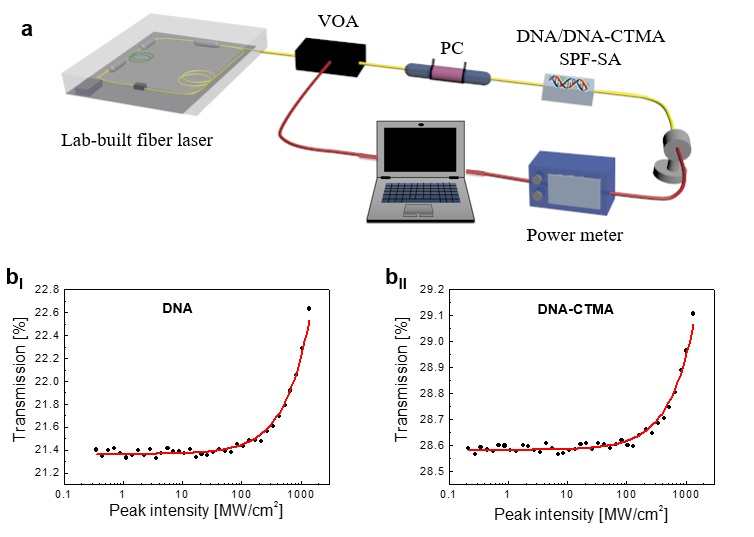
**

**Supplementary Figure 1. (a)** Schematic of the experimental setup for measurement of nonlinear transmission (VOA: variable optical attenuator, PC: polarization controller). **(b)** Intensity-dependent transmission results of the DNA and DNA-CTMA SPF-SA.


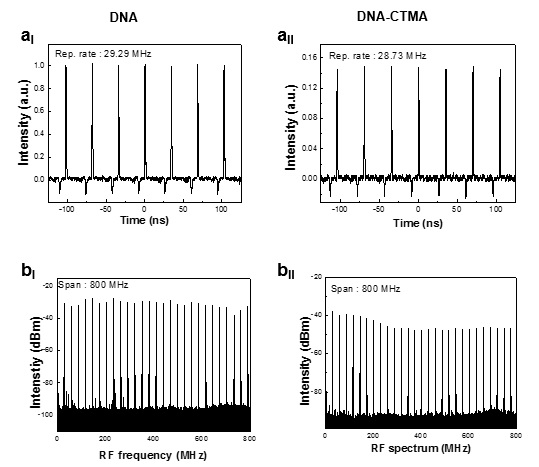


**Supplementary Figure 2.** Laser performance based on DNA and DNA-CTMA SA: **(a)** Oscilloscope measurements of pulse trains. **(b)** Wide-band RF spectra of the output pulse.
